# β-Hairpin of Islet Amyloid Polypeptide Bound to an Aggregation Inhibitor

**DOI:** 10.1038/srep33474

**Published:** 2016-09-19

**Authors:** Ewa A. Mirecka, Sophie Feuerstein, Lothar Gremer, Gunnar F. Schröder, Matthias Stoldt, Dieter Willbold, Wolfgang Hoyer

**Affiliations:** 1 Institute of Physical Biology, Heinrich Heine University Düsseldorf, 40204 Düsseldorf, Germany; 2 Institute of Complex Systems, Structural Biochemistry (ICS-6), Research Center Jülich, 52425 Jülich, Germany

## Abstract

In type 2 diabetes, the formation of islet amyloid consisting of islet amyloid polypeptide (IAPP) is associated with reduction in β-cell mass and contributes to the failure of islet cell transplantation. Rational design of inhibitors of IAPP amyloid formation has therapeutic potential, but is hampered by the lack of structural information on inhibitor complexes of the conformationally flexible, aggregation-prone IAPP. Here we characterize a β-hairpin conformation of IAPP in complex with the engineered binding protein β-wrapin HI18. The β-strands correspond to two amyloidogenic motifs, 12-LANFLVH-18 and 22-NFGAILS-28, which are connected by a turn established around Ser-20. Besides backbone hydrogen bonding, the IAPP:HI18 interaction surface is dominated by non-polar contacts involving hydrophobic side chains of the IAPP β-strands. Apart from monomers, HI18 binds oligomers and fibrils and inhibits IAPP aggregation and toxicity at low substoichiometric concentrations. The IAPP β-hairpin can serve as a molecular recognition motif enabling control of IAPP aggregation.

Aberrant protein aggregation into amyloid fibrils occurs in many age related diseases, including Alzheimer’s disease (AD), Parkinson’s disease (PD), and Type 2 Diabetes (T2D)^1^. In T2D, the 37 amio acid residue polypeptide IAPP aggregates into pancreatic islet amyloid deposits^2^,^3^. IAPP is a hormone stored in β-cell secretory granules and co-secreted with insulin, with putative physiological roles in pancreatic islets as well as in the central nervous system. IAPP can convert into the amyloid state due to the occurrence of three amyloidogenic regions in its amino acid sequence, i.e., IAPP(8–20), IAPP(20–29), and IAPP(30–37)^4^,^5^,^6^,^7^,^8^. The amyloidogenic regions largely adopt β-sheet structure in IAPP amyloid fibrils^9^,^10^. In the amyloid state, IAPP is cytotoxic, contributing to β-cell loss in T2D and to the failure of islet transplantation. Inhibition of IAPP amyloid formation therefore has therapeutic potential, and both polypetide-based and small molecule approaches have provided promising results^11^,^12^,^13^,^14^,^15^. However, monomeric IAPP is intrinsically disordered, rendering structure-based design and optimization of inhibitors diffcult^14^. Furthermore, there is a need of inhibitors that interact with monomers or early oligomers and prevent the formation of any toxic species^2^,^16^.

To provide structural insight into the inhibition of IAPP amyloid formation, we characterize here the interaction of IAPP with the aggregation inhibitor β-wrapin HI18. HI18 is an engineered binding protein obtained from a β-wrapin (β-wrap protein) library by phage display selection against IAPP^17^. The library is based on the scaffold ZAβ_3_, an affibody protein that sequesters a β-hairpin conformation of the Alzheimer’s disease-associated amyloid-β peptide (Aβ)^18^,^19^. HI18 is a homodimer of 58 amino acid-subunits which are linked by a disulfide bond between the Cys-28 residues (Fig. 1a). Compared to ZAβ3, HI18 exhibits two amino acid exchanges per subunit, namely Ala-10 to Glu and Leu-34 to Ile (Fig. 1a). HI18 binds monomeric IAPP with a dissociation constant of 220 nM^17^. We have previously shown that a HI18-IAPP fusion protein has very low aggregation propensity, enabling recombinant production of IAPP^17^. We have moreover described a related, multi-specific β-wrapin, AS10, which inhibits aggregation and toxicity of IAPP as well as Aβ and the Parkinson’s disease-related protein α-synuclein, and which binds presumably all of these three intrinsically disordered proteins in a β-hairpin conformation^20^.

Here, we report the NMR structure of the IAPP:HI18 complex, relate the identified IAPP β-hairpin to previously observed β-hairpins of Aβ^19^ and α-synuclein^21^, and investigate the mechanism of inhibition of IAPP aggregation by HI18.

## Results and Discussion

### Structure of the IAPP:HI18 complex

**Figure 1:**
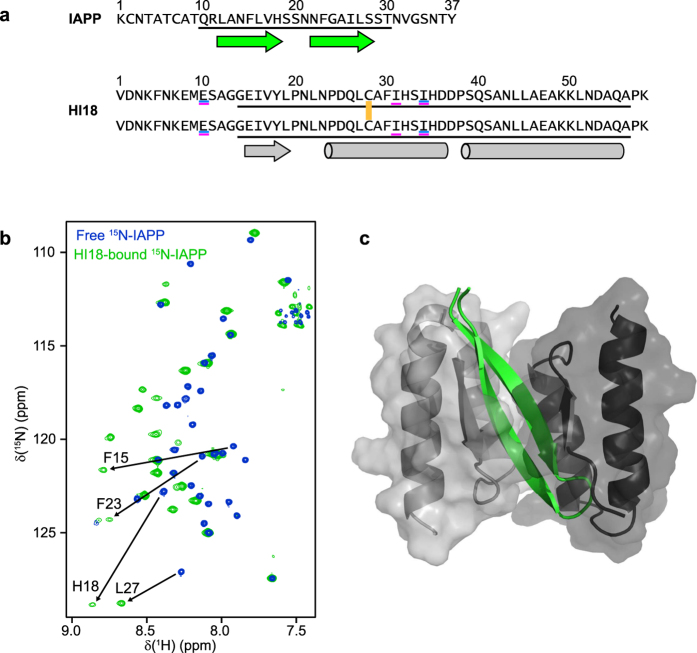
Sequences and topology of the IAPP:HI18 complex. (**a**) Amino acid sequences of IAPP and HI18. The two subunits of HI18 are linked by a disulfide bond involving the Cys-28 residues (yellow). Residues that are exchanged in HI18 compared to ZAβ3 and AS10 are underlined in blue and magenta, respectively. The segments that constitute the folded core of the IAPP:HI18 complex are underlined in black. The positions of α-helical and β-sheet secondary structure in the complex are indicated by cylinders and arrows, respectively. (**b**) (^1^H–^15^N)-HSQC NMR spectra of [*U*-^13^C,^15^N]-IAPP in the absence (blue) and presence (green) of a 20% molar excess of [*NA*]-HI18. Selected resonances experiencing large changes in chemical shifts upon binding are highlighted. (**c**) Ribbon drawing of the IAPP:HI18 complex. Residues 10–30 of IAPP are shown in green. Residues 13–56 of the two HI18 subunits are shown in light and dark gray with semi-transparent surface display.

**Figure 2:**
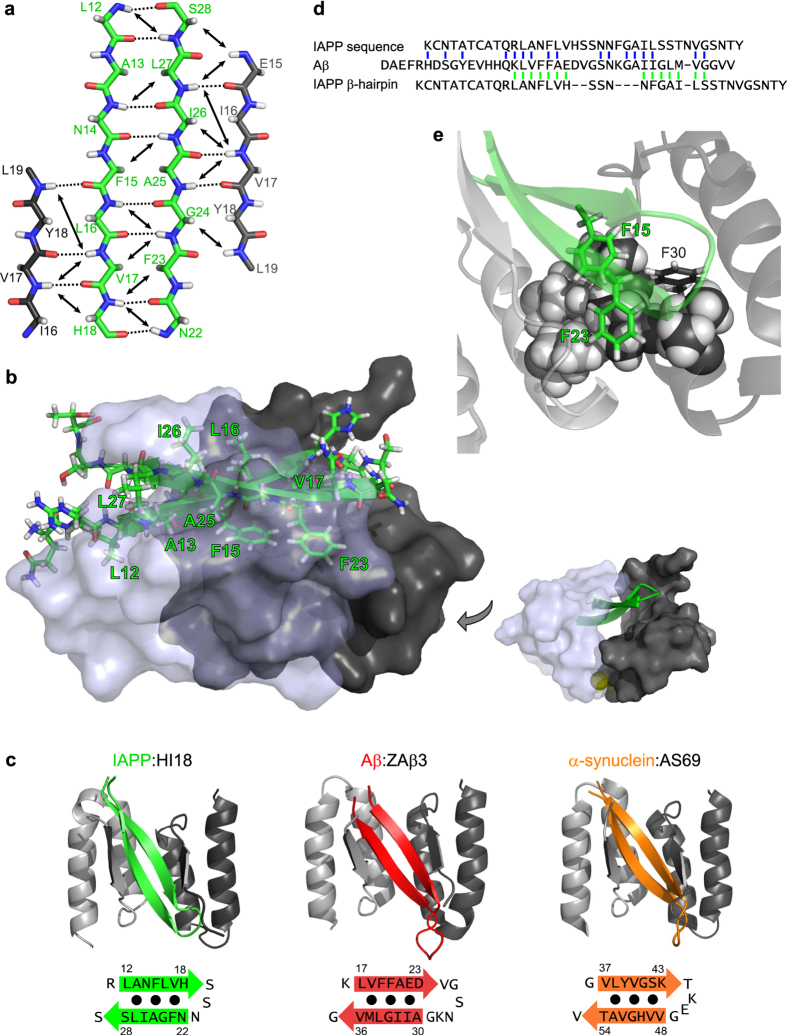
β-Hairpin conformation of IAPP bound to HI18. (**a**) Scheme of the β-sheet registry in the IAPP:HI18 complex. β-Strand backbones of IAPP (green) and the two HI18 subunits (light and dark grey) are displayed in straight extended conformation for clarity. Long-range HN-HN and HN-Hα NOE connectivities defining the β-sheet registry are represented by arrows. Backbone hydrogen bonds identified by PyMOL are shown as dotted lines. (**b**) Ribbon and stick representation of the IAPP β-hairpin (green) interacting with HI18. The two HI18 subunits are shown in semi-transparent and non-transparent surface representation, respectively. Hydrophobic residues in the IAPP β-hairpin are labelled. The sulfur atoms constituting the disulfide bond between the HI18 subunits are displayed in yellow in the image on the right. (**c**) Comparison of the β-hairpin of IAPP (green) bound to HI18 with β-hairpins of Aβ (red) and α-synuclein (orange) bound to the related binding proteins ZAβ3 (PDB:2OTK) and AS69 (PDB:4BXL), respectively. (**d**) Sequence alignment of IAPP and Aβ based on sequence similarity^32^,^33^ (top, blue) or based on the positions of the β-strands in the HI18- and ZAβ_3_-complexes (bottom, green). (**e**) Aromatic-aromatic and aromatic-hydrophobic interactions in the core of the IAPP:HI18 complex. Phe-15 and Phe-23 of IAPP are located in the central core of the complex where they are in contact with each other. One face of both Phe-15 and Phe-23 packs against an extended hydrophobic interaction surface composed of the side chains of Leu-27, Ile-31, and Ile-34 (displayed as spheres) of the two HI18 subunits.

In (^1^H-^15^N) HSQC NMR spectroscopy, free [*U*-^13^C,^15^N]-IAPP exhibits limited resonance dispersion as a consequence of its intrinsically disordered nature (Fig. 1b). Upon addition of [*NA*]-HI18 resonance dispersion increases, with several resonances experiencing a downfield shift into the chemical shift region characteristic of β-sheet conformation, indicating that binding to HI18 is coupled to folding of IAPP. The structure of the IAPP:HI18 complex was determined by high-resolution liquid-state NMR spectroscopy (Fig. 1c and Supplementary Table 1, PDB: 5K5G). The folded core of the complex comprises residues 10–30 of IAPP and residues 14–56 of the HI18 subunits (Fig. 1a). IAPP adopts a β-hairpin conformation upon binding to HI18. The β-hairpin is wrapped by the two HI18 subunits, each contributing two α-helices and a short β-strand that extends the IAPP β-hairpin, giving rise to a four-stranded intermolecular antiparallel β-sheet (Figs 1c and 2a). The IAPP:HI18 complex exhibits C2 pseudosymmetry, with a C2 axis passing through the center of the IAPP β-hairpin and through the Cys-28 disulfide bond that connects the HI18 subunits.

### Two amyloidogenic motifs contribute β-strands to a β-hairpin of IAPP established around serine 20

The IAPP β-hairpin comprises residues 12–28, with a turn around Ser-20 connecting the β-strands 12-LANFLVH-18 and 22-NFGAILS-28 (Fig. 2a). The two β-strands correspond to two of three amyloidogenic regions in IAPP^4^,^5^,^8^. The third amyloidogenic region IAPP(30–37)^6^, in contrast, remains disordered in the IAPP:HI18 complex as judged by its random coil-like secondary chemical shifts and by the absence of NOEs indicative of secondary structure formation. Apart from the backbone hydrogen bonds, interactions of the IAPP β-hairpin are dominated by non-polar contacts between hydrophobic side chains. The side chains of Leu-12, Ala-13, Phe-15, Leu-16, Val-17, Phe-23, Ala-25, Ile-26, and Leu-27 are involved in intramolecular interactions and pack against the tunnel-like binding interface of HI18 (Fig. 2b).

Molecular dynamics simulations have observed significant population of β-hairpin structures in the conformational pool of the intrinsically disordered IAPP monomer, in particular β-hairpins with a turn in the region around Ser-20 and highest β-sheet probability in those sequence regions that also form β-strands in complex with HI18^22^,^23^,^24^. These β-hairpins have been described as the most stable conformations of monomeric, free IAPP^23^. The good agreement of β-hairpin conformations observed in MD simulations of free IAPP with the structure of HI18-bound IAPP suggests that conformational selection may be involved in the binding of HI18 to IAPP. However, the present experimental data does not allow to determine if and to what extent binding is governed by conformational selection.

Interestingly, a high sensitivity of IAPP amyloidogenicity and toxicity to mutations at residues 20, located at the center of the β-hairpin turn, has been reported^25^,^26^,^27^. The Ser-20 to Gly substitution is the only known polymorphism of human IAPP and is associated with earlier onset of T2D. It leads to increased amyloid formation and toxicity^26^,^27^ and has been proposed to support IAPP aggregation by initiating a turn-like structure^28^.

### Aromatic-aromatic and aromatic-aliphatic interactions in the IAPP:HI18 complex

Aromatic-aromatic interactions have been detected in monomeric IAPP^29^, influence the rate of fibril formation^30^, and can play a major role for the inhibition of IAPP aggregation^31^. In the IAPP:HI18 complex, Phe-23 of IAPP is in contact with Phe-15 of IAPP and Phe-30 of one HI18 subunit (Fig. 2e). Phe-15 and Phe-23 are located in the central core of the IAPP:HI18 complex, where they pack against a cluster of hydrophobic side chains of HI18, including one of the Ile-34 residues that were exchanged in HI18 in comparison to the ZAβ3 scaffold sequence.

### Relation of the IAPP β-hairpin to β-hairpins of Aβ and α-synuclein

The structure of IAPP bound to HI18 is very similar to those of Aβ bound to ZAβ_3_ (PDB:2OTK)^19^ and of α-synuclein bound to β-wrapin AS69 (PDB:4BXL)^21^ (Fig. 2c). The corresponding sequence regions that adopt β-hairpin conformation are IAPP(12–28), Aβ(17–36), and α-synuclein(37–54) (Fig. 2c). Despite their structural resemblance the three β-hairpins exhibit only modest amino acid sequence similarity: The β-strands of IAPP show 14% amino acid identity and 21% similarity to those of Aβ and 0% amino acid identity and 29% similarity to those of α-synuclein. Furthermore the length of the turn connecting the β-strands differs among the three proteins.

For the overall amino acid sequences a comparatively high similarity between IAPP and Aβ has been noted (24% identity and 47% similarity)^32^,^33^. Interestingly, the sequence alignment based on the positions of the β-strands in the HI18- and ZAβ_3_-complexes does not match the alignment based on sequence similarity (Fig. 2d). Obviously, the sequence features determining the common adoption of the β-hairpin conformation are not directly reflected in the sequence similarity alignment.

There is increasing evidence for molecular links between different protein aggregation diseases. For example, Aβ plaques frequently occur in Lewy body disease while α-synuclein Lewy bodies are a feature of most of the AD cases^34^,^35^, and α-synuclein cross-seeds tau aggregation^36^. Epidemiological studies show an increased risk of AD in people with diabetes^37^. Intravenous injection with preformed Aβ fibrils into human IAPP transgenic mice triggered IAPP amyloid formation in the pancreas, and IAPP was detected in cerebral and vascular Aβ deposits in brains of AD patients^38^,^39^. IAPP and Aβ moreover interact at the pre-fibrillar stage by forming hetero-oligomers, a reaction affecting the formation of homo-oligomers and homo-fibrils of both polypeptides^40^,^41^. Interestingly, the regions of IAPP and Aβ involved in hetero-oligomer formation are in good agreement with those forming β-hairpins in complex with HI18 and ZAβ3, respectively^32^. Thus, it will be interesting to test the hypothesis that hairpin structures such as those displayed in Fig. 2c, which present largely hydrophobic surfaces, are involved in hetero-oligomer formation, in analogy to the previously reported formation of Aβ homo-oligomers containing β-hairpins^42^,^43^.

### HI18 inhibits IAPP aggregation at substoichiometric concentrations

**Figure 3:**
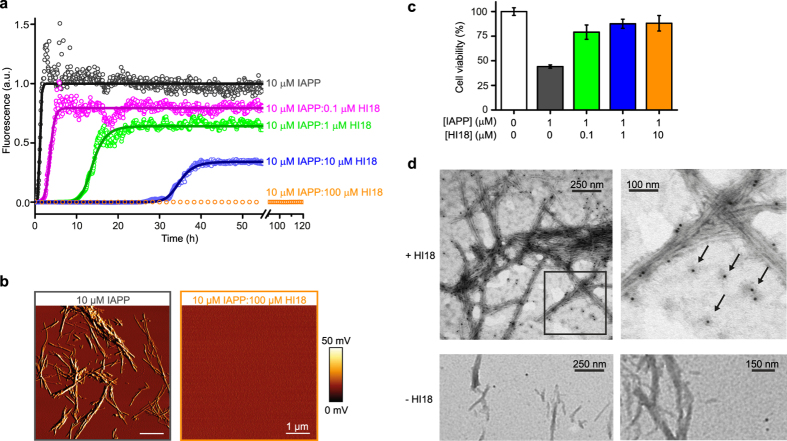
HI18 inhibits amyloid formation and toxicity of IAPP at low substoichiometric concentrations. (**a**) ThT time course of amyloid formation of IAPP in the absence and presence of HI18 (circles). To obtain half-times of aggregation (t_1/2_) the data was fitted to a model of secondary nucleation dominated aggregation kinetics using the program AmyloFit^55^,^56^ (solid lines). (**b**) AFM amplitude images of IAPP in absence and presence of HI18 at the end of the aggregation experiment in (**a**). (**c**) MTT assay to determine the viability of human 1.1B4 pancreatic islet cells upon addition of IAPP aged in the absence or presence of HI18. The data are representative of experiments carried out in quadruplicate (mean ± s.d.), expressed as percentage relative to the untreated cells (control). (**d**) Interaction of HI18 with IAPP oligomers and fibrils analyzed by TEM with immunogold labeling detecting specifically HI18. Top panels, HI18 was added to a sample withdrawn during the growth phase of an IAPP aggregation reaction proceeding in the absence of HI18, and detected by immunogold-labeling. The image on the right is a magnification of the boxed area in the left image. Bottom panels, control samples in which HI18 addition was omitted, confirming the absence of unspecific immunogold labeling of IAPP aggregates.

The inhibition of IAPP aggregation by HI18 was studied by a Thioflavin T fluorescence assay and atomic force microscopy (AFM). In the absence of HI18, the aggregation of a solution of 10 μM IAPP occurred with a lag time of only a few minutes and a half-time (t_1/2_) of 1.2 h (Fig. 3a), yielding IAPP amyloid fibrils (Fig. 3b). Addition of HI18 led to a concentration-dependent inhibition of aggregation, with a prolongation of the half-time of aggregation by 3-fold (t_1/2_ = 3.6 h), 12-fold (t_1/2_ = 14 h), and 28-fold (t_1/2_ = 35 h) in the presence of a 1:100, 1:10, and 1:1 HI18:IAPP molar ratio, respectively (Fig. 3a). At a 10:1 HI18:IAPP molar ratio, no increase in Thioflavin T fluorescence was detected during a 5-day experiment, and no amyloid fibrils or other IAPP aggregates were discernible by AFM (Fig. 3a,b).

To assess the impact of HI18 on IAPP toxicity in a pancreatic islet cell culture system, we evaluated the viability of human 1.1B4 cells^44^ in the absence and presence of HI18. When IAPP was aged at a concentration of 10 μM for 1 hour and diluted into the cell culture medium to a final concentration of 1 μM, a significant decrease in cell viability was observed in an MTT assay (Fig. 3c), in agreement with cytotoxicity of IAPP oligomers and/or fibrils^45^. However, when IAPP was aged in the presence of HI18, the cell viability was largely rescued, demonstrating that HI18 inhibits toxic aggregate formation (Fig. 3c).

The potent aggregation inhibition at substoichiometric HI18 concentrations reveals that HI18 interferes with IAPP aggregation not only by removal of IAPP monomers from the aggregation reaction. In addition, HI18 must exert an activity which effectively impedes fibril nucleation or elongation. Substoichiometric concentrations of HI18 inhibited IAPP aggregation also in seeded aggregation assays, in which the primary nucleation step of the aggregation reaction is bypassed by the addition of preformed fibril seeds (Supplementary Fig. 1). This demonstrates that HI18 affects at least one of the reaction steps subsequent to primary nucleation, i.e. secondary nucleation or elongation^46^. Interference with fibril nucleation or elongation at substoichiometric concentrations requires a high-affinity interaction with oligomeric or fibrillar species. We could not observe such interactions during the lag-phase of aggregation due to the absence of detectable amounts of higher-order assemblies (Fig. 3b). Therefore, we enriched IAPP oligomers and fibrils by permitting aggregation to proceed in the absence of HI18, added HI18 when approximately the middle of the growth phase was achieved, and analyzed the binding of HI18 to IAPP aggregates by HI18-targeted immunogold-labeling and transmission electron microscopy (TEM) (Fig. 3d). HI18 was indeed localized on fibril surfaces, where it could potentially interfere with secondary nucleation (Fig. 3d, upper panels). In addition, HI18 was associated with smaller assemblies, which might be involved in primary nucleation (Fig. 3d, arrows). Due to limited resolution and contrast, however, we cannot exclude that these smaller assemblies represent aggregates other than IAPP oligomers. Enrichment of HI18 at fibril ends, in line with a potential effect on fibril elongation, was not evident from the TEM images. When HI18 addition was omitted nanogold particles were rarely observed and did not co-localize with IAPP aggregates, demonstrating the absence of unspecific immunogold labeling of IAPP aggregates (Fig. 3d, bottom panels).

A possible explanation for the potent interference of HI18 with the nucleation of aggregation would be a high-affinity interaction of HI18 with β-sheets present in intermediates on the IAPP aggregation pathway. The formation of intermediate β-sheet structures has in fact been implicated in the early steps of the amyloid formation reaction: the population of β-hairpin conformations in the monomeric state correlates with the amyloidogenicity of IAPP variants, and dimer formation by β-hairpin stacking has been suggested to occur on-pathway based on MD simulations^22^,^24^,^47^. Moreover, two-dimensional IR spectroscopy detected an initial formation of β-sheet secondary structure exactly in those regions of the IAPP sequence that also form β-strands in the HI18-complex^48^.

## Conclusions

This study identifies a β-hairpin conformation of IAPP that is accessible for molecular recognition. It demonstrates how the conformational preferences of IAPP can be targeted to achieve inhibition of amyloid formation. Comparison with previously observed structures of bound Aβ and α-synuclein highlights common β-hairpin molecular recognition motifs which exhibit only modest sequence similarity. The β-hairpin might represent a favorable compact motif for molecular recognition also by other classes of molecules aiming at IAPP inhibition. The β-wrapin HI18 acts as an exceptionally potent aggregation inhibitor, suggesting that these principles should be further exploited in future development of inhibitors of amyloid formation.

## Methods

### Preparation of IAPP

To prepare monomeric starting material, synthetic human IAPP (EMD Millipore) amidated at the C-terminus was dissolved in 6 M guanidine hydrochloride (GdnHCl), 20 mM sodium phosphate, pH 6.0 and loaded onto a Superdex 75 10/300 GL column (GE Healthcare) equilibrated in 20 mM sodium phosphate, pH 6.0. Monomeric IAPP eluted at a volume of ~17 mL and was stored at −80 °C. For NMR experiments, recombinant human IAPP was produced using a cleavable fusion construct as described^17^.

### Preparation of β-wrapin HI18

HI18 was selected from a β-wrapin phage display library as described^17^. Protein expression was carried out using a pET302/NT-His vector encoding HI18 with an N-terminal His_6_-tag in *E. coli* BL21(DE3) cells in LB medium. Expression was induced with 1 mM IPTG at OD 0.6–0.8 followed by overnight incubation at 27 °C. The cell pellet was resuspended in 20 mM imidazole, 500 mM NaCl, 20 mM sodium phosphate, pH 8.0, containing EDTA-free protease inhibitors (Roche Applied Sciences) and lysed by a cell disrupter (Constant Systems). Insoluble material was removed by centrifugation at 28,000 × *g* and the supernatant was loaded on a HisTrap FF 5 ml column (GE Healthcare). The dimeric form of HI18 was collected from a HiLoad 16/60 Superdex 75 size-exclusion chromatography column (GE Healthcare) in 20 mM sodium phosphate, pH 6.0. For NMR experiments, HI18 was expressed in M9 minimal medium supplemented with ^15^N-NH_4_Cl (1 g/l) and ^13^C_6_-glucose (2 g/l) and purified as above.

### NMR and structure determination

The NMR spectra were acquired at 25 °C using VNMRS instruments (Varian) at proton frequencies of 800 and 900 MHz, each equipped with a cryogenically cooled Z-axis pulse-field-gradient (PFG) triple resonance probe. NMR samples contained [*U*-^13^C,^15^N]-IAPP or [*U*-^13^C,^15^N]-HI18 at a concentration of 0.4 mM, a 20% molar excess of the respective non-isotopically enriched binding partner and 7% D_2_O in 20 mM sodium phosphate, pH 6.0. NMR samples of the IAPP:HI18 complex exhibited only a slight loss of signal intensity over the course of several weeks, reflecting the potent inhibition of IAPP aggregation by HI18 under these conditions (Shigemi tubes, T(measurement) = 25 °C, T(storage) = 4 °C). NMR data were processed using NMRPipe^49^ and analyzed with CcpNmr^50^. Backbone assignments were obtained using BEST-TROSY experiments^51^ and side-chain assignments were obtained using standard triple resonance heteronuclear NMR techniques. Nuclear Overhauser effect (NOE) based distance restraints for structure calculation were derived from 3D (^1^H-^1^H-^15^N)-NOESY-HSQC (120 ms mixing time) and (^1^H-^13^Cali-^1^H)-HSQC-NOESY (100 ms mixing time) experiments. Prediction of protein backbone angles from chemical shifts was performed with DANGLE^52^.

Structure calculations based on NOE distance restraints and dihedral angle restraints were accomplished with a modified version of CNS v. 1.2.1^53^,^54^ using an optimized version of the PARALLHDG force field. The MD protocol contained 30 ps high temperature torsion angle dynamics (10,000 K) and 20,000 steps during two cooling phases (2,000 K and 50 K). Ten lowest energy structures (overall CNS energy) out of 100 calculated were selected and deposited (PDB:5K5G). 90.0% of the residues are found in the core regions, 9.9% in the allowed regions and 0.1% in the generous regions of the Ramachandran diagram. All structural figures were generated using PyMOL Molecular Graphics System (Schrödinger).

### Aggregation assay

Fluorescence was monitored in samples containing HI18 and IAPP at indicated concentrations in 20 mM sodium phosphate, pH 6.0 containing 40 μM Thioflavin T (ThT) in a final volume of 100 μl in a round-bottom 96-well black plate (Nunc). Aggregation was performed at 28 °C on an Infinite M1000 plate reader (Tecan). Fluorescence was measured at 490 nm with an excitation Wavelength of 450 nm. To obtain half-times of aggregation the data was fitted to a model of secondary nucleation dominated aggregation kinetics using the program AmyloFit^55^,^56^.

### Atomic force microscopy (AFM)

50 μl of 10 μM solutions of IAPP aggregated in presence and absence of HI18 were adsorbed for 1 h to a freshly cleaved mica surface followed by washing with milliQ water and drying with a gentle stream of N_2_ gas. Imaging was performed under air dried conditions in intermittent contact mode in a JPK Nano Wizard II atomic force microscope using a silicon cantilever with silicon tip (OMCL-AC160TS, Olympus) with a typical tip radius of 9 ± 2 nm, a force constant of 42 N/m and a line rate of 0.5 Hz. The images were processed using JPK Data Processing software.

### MTT cell viability assay

The viability of a human pancreatic beta cell line in the presence of IAPP and HI18 was tested with an MTT assay (Cell Proliferation Kit I, Roche Diagnostic). 1.1B4 cells (purchased from Public Health England) were seeded in 96-well tissue culture plate at a density of 10,000 cells/well in 100 μl of media (RPMI-1640, 10% fetal calf serum) and incubated for 24 h. Protein samples were aged under amyloid formation conditions as described above for 1 h at an IAPP concentration of 10 μM and diluted into the cell culture medium to a final concentration of 1 μM (the concentration of HI18 in the samples is given in Fig. 3c). Following application of the protein samples, cells were further incubated for 24 h. To assess the effects of test samples on the cells, MTT was added to the cells at a final concentration of 0.5 mg/ml in PBS followed by incubation for an additional 4 h at 37 °C. Next, 100 μl of the solubilization solution (DMSO) was added and the absorbance at 590 nm was measured with 620 nm as a reference wavelength in an Infinite M1000 plate reader (Tecan). The data was normalized to the value of untreated control cells. All cell cultures were maintained in a 5% CO_2_ humidified atmosphere at 37 °C.

### Immunogold labeling and transmission electron microscopy

A sample of IAPP at a corresponding monomer concentration of 25 μM was withdrawn during the growth phase of aggregation and incubated for 1 h after addition of HI18 at a final concentration of 2.5 μM. For immunogold-labeling of β-wrapin HI18, protein sample was adsorbed on formvar-carbon coated copper grid (Plano) for 30 min. After blocking using 5% (w/v) BSA in PBS for 60 min, grids were incubated with goat anti-affibody antibody (Abcam) which recognizes HI18 as proved in preceding experiments, diluted 1:200 in 1% (w/v) BSA in PBS for 1 h. After being washed six times in PBS, grids were incubated with gold-labeled rabbit anti-goat IgG (Abcam, 10-nm gold) diluted 1:20 in 1% (w/v) BSA in PBS for 1 h, followed by six washes in PBS. Postfixation was done with 1% (w/v) glutaraldehyde in PBS for 5 min. After excessive washing in water, the sample was negatively stained using 2% (w/v) uranyl-acetate in water for 5 min. Analysis was performed on a Zeiss EM 902A transmission electron microscope operated at 80 kV.

## Additional Information

**Accession codes**: The structure of the IAPP:HI18 complex has been deposited in the Protein Data Bank as entry 5K5G. Chemical shifts and NMR restraints of the IAPP:HI18 complex have been deposited in the Biological Magnetic Resonance Bank under accession code 30099.

**How to cite this article**: Mirecka, E. A. *et al*. β-Hairpin of Islet Amyloid Polypeptide Bound to an Aggregation Inhibitor. *Sci. Rep.* **6**, 33474; doi: 10.1038/srep33474 (2016).

## Supplementary Material

Supplementary Information
