# Supplementary material for: β-Hairpin of Islet Amyloid Polypeptide Bound to an Aggregation Inhibitor
Source: Sci Rep. 2016 Sep 19;6:33474. doi: 10.1038/srep33474 (PMC5027568; doi:10.1038/srep33474)
Supplement: Supplementary Information [file srep33474-s1.pdf]

Supplementary Information

β-Hairpin of Islet Amyloid Polypeptide Bound to an Aggregation Inhibitor

Ewa A. Mirecka, Sophie Feuerstein, Lothar Gremer, Gunnar F. Schröder, Matthias Stoldt, Dieter Willbold & Wolfgang Hoyer

Content

- Supplementary Figure 1: Inhibition of seeded IAPP aggregation by substoichiometric concentrations of HI18.
- Supplementary Table 1: NMR and refinement statistics of the IAPP:HI18 complex

Supplemental Figure 1: Inhibition of seeded IAPP aggregation by substoichiometric concentrations of HI18.

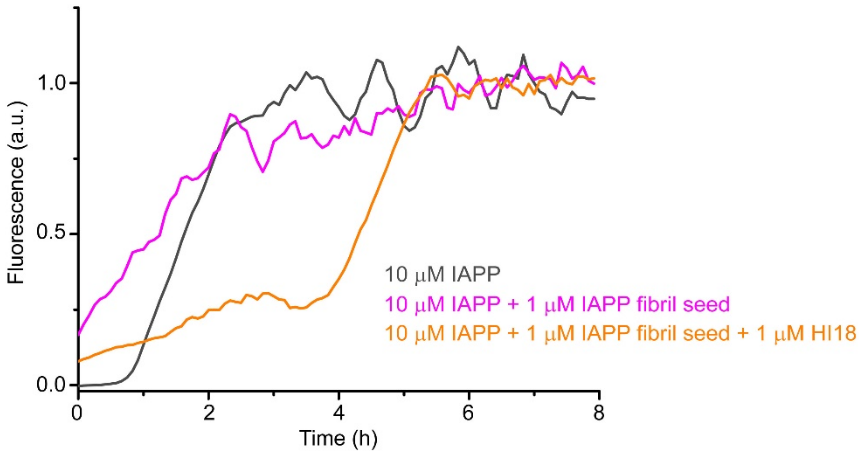

ThT time traces of amyloid formation in 10 μM solutions of monomeric IAPP are shown. In contrast to unseeded amyloid formation (grey time trace), seeded amyloid formation of IAPP (magenta time trace) occurs without a discernable lag-time as the primary nucleation step of amyloid formation is bypassed. However, in the presence of a substoichiometric amount of HI18 (orange time trace), seeded aggregation is strongly retarded, with an increase in the half-time of aggregation by ~3-fold at a HI18:IAPP molar ratio of 1:10.

Supplementary Table 1: NMR and refinement statistics of the IAPP:HI18 complex

NMR distance & dihedral constraints	Number
Distance constraints	
Unambiguous NOE constraints	1235
Intra-residue	554
Inter-residue	681
Sequential ($\|i-j\| = 1$)	293
Medium-range ($\|i-j\| \leq 5$)	172
Long-range ($\|i-j\| > 5$)	104
Intermolecular	112
Ambiguous NOE constraints	281
Total dihedral angle restraints	186

Structure statistics	Value
Violations	
Distance constraints (Å)	0.025 ± 0.001
Dihedral angle constraints (º)	0.93 ± 0.12
Max. distance constraint violation (Å)	0.29
Deviations from idealized geometry	
Bond lengths (Å)	0.0046 ± 0.0002
Bond angles (º)	0.58 ± 0.02
Impropers (º)	1.61 ± 0.06
Average pairwise r.m.s. deviation* (Å)	
Heavy	0.95 ± 0.07
Backbone	0.58 ± 0.07

*Pairwise r.m.s. deviation was calculated among 10 refined structures
